# Emerging Nutritional Problem of Adult Population: Overweight/Obesity and Associated Factors in Addis Ababa City Communities, Ethiopia—A Community-Based Cross-Sectional Study

**DOI:** 10.1155/2020/6928452

**Published:** 2020-10-19

**Authors:** Tsedeke Wolde Hailemariam, Samrawit Solomon Ethiopia, Andamlak Gizaw Alamdo, Haimanot Ewnetu Hailu

**Affiliations:** Department of Public Health, St. Paul's Hospital Millennium Medical College (SPHMMC), Addis Ababa, Ethiopia

## Abstract

**Background:**

Obesity is an emerging public health problem in developing countries. There is limited study conducted in Ethiopia to determine the prevalence of obesity and its associated factors among adult population. Therefore, this study aimed at determining the prevalence of overweight/obesity and the associated factors among adults aged 25–64 years in Addis Ababa city community residents, Ethiopia.

**Methods:**

A community-based cross-sectional study was conducted from April 10, 2017, to May 20, 2017, in Addis Ababa. A total of 512 adults were recruited. A two-stage cluster followed by a systematic random sampling technique was used for sample selection. Data were collected using questionnaires and anthropometric measurements. The adjusted odds ratio (AOR) with a 95% CI was reported to show the strength of association. A *P* value < 0.05 was considered statistically significant.

**Results:**

A total of 484 adults participated in the study with a response rate of 94.5%. The prevalence of overweight and obesity among study participants was found to be 99 (21.5%) and 14 (2.9%), respectively. Males were 90% less likely to be obese when compared to females (AOR = 0.10 (95% CI: 0.01–0.84)). Illiterate people were 94% less likely to be obese compared to those who were literate people (AOR = 0.06 (95% CI: 0.01–0.44)). Nonhypertensive individuals were 86% less likely to be obese when compared to hypertensive (AOR = 0.14 (95% CI: 0.03–0.69)).

**Conclusion:**

The combined prevalence of overweight and obesity was found to be considerably high in Addis Ababa city residents compared to the national figure. Being female, literate, and presence of hypertension are independent predictors of overweight/obesity in the study population. Thus, the concerned bodies should initiate efforts to tackle the newly emerging public health problem of the country and promote healthy lifestyle behaviors in the inhabitants of city settings.

## 1. Background

Overweight and obesity are now stepping in to the borders of developing countries. Countries with a drastic shift from a state of undernutrition to overnutrition are facing chronic nutrition-related noncommunicable diseases (NCDs) such as cancer, hypertension, cardiovascular disease, and type two diabetes mellitus [1]. Various studies have shown that obesity and nutrition transition have been evident even in middle- and low-income countries of urban areas of sub-Saharan Africa. Following the nutrition transition and epidemiologic transition, chronic noncommunicable diseases are prevailing in the developing countries [2].

Most countries in Asia, Latin America, Northern Africa, the Middle East, and the urban areas of sub-Saharan Africa have all experienced a shift in the overall structure of their dietary pattern with related disease patterns over the last few decades. Major dietary change includes a large increase in the consumption of fat and added sugar in the diet, often a marked increase in animal food products contrasted with a fall in total cereal intake and fiber [3].

The obesogenic environment as evidenced by the consumption of high-energy-density diets and diets low in complex carbohydrates and a sedentary life style are known to be among the contributors to the developing problem of obesity [4]. The globalization of marketing, the emergence of new technologies, the international communication systems, and the access to transportation have been a potential for low- and middle-income countries to be victims of obesity and the associated problems at a faster rate [4].

Even over the last 30 years, nutrition study in Ethiopia depicted that the urban population of Ethiopia are at risk of obesity. “Some groups of urban Ethiopians are in a phase of change of socioeconomic status, dietary habits, and physical work outputs towards a more Western pattern” [5]. The rise in obesity prevalence represents a challenge for the health care system, which is traditionally overburdened by underweight problems arising from famine, food insecurity, and infectious diseases [6], but now has to cope with obesity-related NCDs which are estimated to account for 46% of all deaths by 2030 [7].

Many years ago, overweight and obesity were not an emerging problem in Ethiopia. However, nowadays, the level of overweight and obesity among adult population increased from 3% in 2000 to 8% in 2016 [8, 9]. Similar studies showed that the magnitude of overweight ranges from 16.1% to 25.3% and that of obesity ranges from 5.6% to 16.2% [10–13]. A study conducted in Tanzania revealed that the prevalence of overweight and obesity among adults was 24.1% and 19.2%, respectively [14]. A similar study conducted among the urban residents of adult Malawians showed that the prevalence of overweight/obesity was found to be 28.1% [15].

In local studies conducted in different Ethiopian cities, the overall prevalence of overweight/obesity among adults in Gonder, Addis Ababa, Dessie, Hawassa, and Bahir Dar are found to be 48.6%, 35.9%, 28.5%, 28%, and 11.3%, respectively [11, 13, 16–18]. Another similar study conducted among permanent employees of the Commercial Bank of Ethiopia and teachers in the government schools of Addis Ababa found that 24.7% of men and 25.7% of women were overweight, and 2.1% men and 10.2% women were obese [19].

Few surveys have been conducted in Addis Ababa city to determine the magnitude of overweight/obesity, and its associated factors among adults aged 18–65 years have not yet been documented for this capital, and there is scarce information about the prevalence and determinants of overweight/obesity. Although there are circumstantial lines of evidence on the increasing level of obesity in the urban population of Ethiopia, there are no recent population-based studies which estimated its prevalence and associated factors given the specific cultural and life style milieu of the urban community in Ethiopia in general and in Addis Ababa city in particular.

The findings of this study can benefit the policy makers to design appropriate interventions in order to tackle the emerging problem of overweight/obesity and its associated factors among the study population. Generally, the study will provide preliminary information for further researches in the study area of obesity and its related noncommunicable diseases.

## 2. Methods

### 2.1. Study Setting, Design, and Period

The study was conducted in Addis Ababa city community residents which is the capital city of Ethiopia. The city is divided into 10 administrative subcities and 116 administrative woredas/districts. It is the largest city in Ethiopia with a population of 3,384,569 people and annual growth rate of 3.8 people. The total population between age groups 15 and 64 years is 2,228,510 [20]. A community-based cross-sectional study was employed from April 10, 2017, to May 20, 2017.

### 2.2. Population and Sampling

The source population was all adult men and women aged 25–64 years who are living in Addis Ababa city during the study period. While the study population was all adult men and women aged 25–64 years who are living in Addis Ababa City for two years or longer, they were eligible to participate in the study. Pregnant women, mothers who were less than two-month postdelivery, women who were on hormonal contraception, and participants with physical deformity, edema, or wasting syndrome were excluded from the study.

The sample size was calculated using the formula for estimation of a single population (*n* = ((*Z*(1−*α*/2))2. *p*(1−*p*))/d2) with the following assumption: *z* = standard normal variable at 95% (1.96) level; *p* = overall prevalence of overweight/obesity among adult population was 28% in Hawassa town [17]; *d* = 5% margin of error; the design effect number is multiplied by 1.5 (De) and added to 10% of nonresponse rate. Hence, the minimum sample size for the study was 512.

A two-stage cluster sampling method was used for sample selection. The primary sampling units were subcities randomly selected from each of the ten study subcities. A total of three subcities were selected from the ten subcities. Secondary sampling units were districts randomly selected from subcities selected for the study. Two districts were randomly selected from each of the study three subcities, and a total of six districts were selected for the study. A systematic sampling was used to identify the households selected for the study. The sample size was drawn from each district was proportional to the population size of the district. Within the selected households, lottery method was used to identify the study participants, and only one sample was drawn from each household.

### 2.3. Data Collection Procedures and Measurements

A structured questionnaire was used to collect sociodemographic (age, sex, occupation, marital status, level of educational, monthly income, wealth index, and family size), life style (history of tobacco and drug use, alcohol consumption, history of hypertension, history of diabetes mellitus, and status of physical activity), anthropometric measurements (weight and height), and assessment of dietary intake. A standardized questionnaire designed by WHO steps instruments for chronic disease risk surveillance questionnaire was used after minor modifications [21]. All the data was collected using this questionnaire by face-to-face interview.

Dietary diversity data was collected using a modified individual dietary diversity questionnaire as recommended by Food and Agricultural Organization (FAO) 2008 [22]. Dietary diversity was assessed by asking respondents to report the different food groups they consumed over the past 24 hours, and the tercile and the mean dietary diversity score of the group was calculated and used in the analysis. Dietary diversity was a diverse food composition which included consumption of all the food categories. This category was divided into three low (<3), medium (4–6), and high (≥7) dietary diversity scores [22].

Weight and height are the main anthropometric measurements that were used in this study. Weight was measured to the nearest 0.1 kg without shoe and wearing light clothing. Height was measured by positioning the subject in the Frankfort plane using a stadiometer. Study participants were in barefoot while measuring their height. Body mass index (BMI) was calculated as weight in kilograms divided by height in meters squared (kg/m^2^) [23]. The overweight is set between a BMI of 25 and 29.99 kg/m^2^ and obese is defined as a BMI ≥30 kg/m^2^ [23]. The outcome variable was dichotomized as overweight and obese (yes) compared to the reference of normal and underweight groups (no).

### 2.4. Data Quality Control

A 5% of pretest of the sample size was carried out to check for validity and the reliability of the questionnaire before the commencement of the main data collection and it was found to be valid and reliable for the study participants. People who were included in the pretest are not part of the main study. Data collectors are thoroughly being trained and briefed about the data collection so that they can come up with valid data. The anthropometric measurement was conducted by diploma nurses. There is a continuous supervision during the data collection.

### 2.5. Data Management and Analysis

Study questionnaires were kept in binders during interview process; all questionnaires collected each day were packed in an envelope and submitted to supervisor's review and feedback. All reviewed and completed questionnaires were stored under lock in canvas bag and kept with the supervisors. Upon completion of the data collection, all filled questionnaires were submitted to the principal investigator (PI). Principal investigator and supervisors were once again checking the questionnaires for completeness and consistency and identify any incomplete questionnaires.

Epi Info data entry template was developed by PI. Then two data encoders (B.S. degree in computer science and data entry experience using Epi Info data and SPSS) were trained for two days on data entry template by PI. Then, data was entered by the two data personnel who were entering independently in Epi Info data-based data entry template (double data entry). Then, the PI has carried out data cleaning. Then, questionnaires of cases with discrepancies were used to check the points of discrepancy and correct according to the data recorded on the questionnaire till all discrepancies were cleaned. Then, the cleaned data set was exported to SPPS.

Data was entered, cleaned, and analyzed using Epi Info version 7 and SPSS version 23. All categorical variables were analyzed using frequencies and percentages. Cross tabulations and Pearson's chi-square test were used to obtain the associations and strength of relationship between the independent and the dependent variables. Logistic regression analysis was used to control for confounding factors. In this model, the dependent variable was BMI while the independent variables were factors that showed statistical significance on chi-square test and on univariate analysis including age, sex, marital status, level of education, occupation, wealth index, morbidity status, dietary diversity score, and physical activity level. A *P* value of ≤ 0.05 was considered significant. The crude odds ratio (COR) and adjusted odds ratio (AOR) with a 95% CI were reported to show the strength of association. A *P* value < 0.05 was considered statistically significant.

### 2.6. Ethical Issues

Ethical clearance was obtained from the Institution Review Board (IRB) of the St. Paul's Hospital Millennium Medical College (SPHMMC). Every study participant was briefed about the research intent and was asked for permission to participate on his or her own will and was informed that he or she was not missing anything for the denial to participate in the study. Informed consent was obtained from all participants in the study. The participants were reassured about confidentiality of the data.

## 3. Results

### 3.1. Characteristics of Study Participants

A total of 484 participants were involved in this study from 512 participants planned to be included in the study, giving a response rate of 95%. From the 484 participants, 253 (52.3%) were men and 231 (47.7%) were women, giving a men to women ratio of 1:0.9. The mean (±SD) age of the study participants was 35.19 (±8.76) years. More than half of the study participants, 268 (55.4%), were under the age of 35 years. From the study participants, 361 (74.6%) were Orthodox, and 71 (14.7%) were Protestants. The majority of the study subjects, 259 (53.5%), were Amhara, followed by Oromo 117 (24.2%) and Tigre 45 (9.3%) (Table 1).

The majority of the study participants, 337 (69.6%), had completed or were attending college or university education and 16 (3.3%) of the study subjects had no formal schooling. Fifty-nine (12.2%) of the study subjects have completed or attended high school education and the rest 72 (14.9%) had junior- or elementary-level education. More than half, 268 (55.4%), of the study participants were married, and 184 (38%) were single. Around one-third, 179 (37%), of the study participants were government employees. Housewives accounted for 37 (7.6%) and merchants accounted for 146 (30.2%) (Table 1).

Around three-fourths, 344 (71.1%), of the study participants had four-person family size. The mean (±SD) monthly income of the study participants was 8154.53 (±5502.31) Ethiopian birr per month. More than half, 277 (57.2%), of the study participants have earned less than or equal to 8000 Ethiopian birr per month. While from the study participants, 170 (35.1%) were of low-tercile or socioeconomic status (SES) of households, and 147 (30.4%) were of high-tercile households (Table 1).

### 3.2. Prevalence of Overweight and Obesity and Their Sociodemographic Association

**Figure 1:**
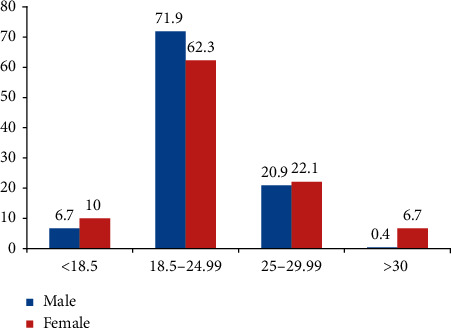
Classification of nutritional status using body mass index (BMI) among adults in Addis Ababa, Ethiopia.

The mean (±SD) body mass index (BMI) of the study participants was 23.06 (±3.35) kg/m^2^. The median with range of BMI was 23.03 (15.43–36.85) kg/m^2^. According to WHO adult BMI classification, the combined prevalence of adult overweight and obesity was 24.4%, the specific prevalence being 2.9% and 21.5% for obesity and overweight, respectively (Figure 1).

The obesity prevalence was higher in women than men having a value of 13 (5.6%) for women and one (0.4%) for men. The same thing was also true for overweight; 22.1% prevalence was seen in women and 20.9% in men (Figure 1).

Sex and obesity were associated in the study population (*P*=0.01) (Table 2). The prevalence of obesity was higher in older age groups when compared to younger groups. The highest prevalence of obesity was observed in the age group of 45–54 years being 9.5%. The same thing was also true for overweight; the highest prevalence of overweight was observed in the age group of 45–54 years being 46.2% (Table 2). The prevalence of obesity was highest in the merchants with the value of 9 (6.2%) followed by housewives accounting for 2 (5.4%). The lowest obesity level was seen in non–government employees which was one (1.3%). The prevalence of obesity was highest among married people with the value of 10 (3.7%). The prevalence of obesity was higher among those of the highest wealth tercile households (6.2%) when compared to the lowest wealth tercile households (0.6%). Family size and monthly income had no statistically significant association with obesity (*P* > 0.05) (Table 2).

Sex and obesity were associated in the study population (*P* < 0.01) (Table 2). The prevalence of obesity was higher in older age groups when compared to younger groups. The highest prevalence of obesity was observed in the age group of 45–54 years being 9.5%. The same thing was also true for overweight; the highest prevalence of overweight was observed in the age group of 45–54 years being 46.2% (Table 2). The prevalence of obesity was highest in the merchant with the value of 9 (6.2%) followed by housewives accounting for 2 (5.4%). The lowest obesity level was seen in non-government employees which was one (1.3%). The prevalence of obesity was highest among married people with the value of 10 (3.7%). Prevalence of obesity was higher among those of the highest wealth tercile households (6.2%) when compared to the lowest wealth tercile households (0.6%). Family size and monthly income had no statistically significant association with obesity (*P* > 0.05) (Table 2).

With regard to the educational status, obesity was seen to be higher among those who had not attended formal school 4 (25%) followed by college degree 9 (2.7%) and high school 1 (1.7%). There was statistically significant association between the two variables (*P* < 0.01) (Table 3).

Majority of the study participants were in the highest tercile of the dietary diversity score 349 (72.1%) while only 36 (7.4%) were in low tercile. When obesity was analyzed with regard to dietary diversity score, 12 (3.4%) were obese among those who were in the highest tercile of dietary diversity score. Two (2%) were in the medium tercile, and none of them were in the low tercile of the dietary diversity score, but there was no association detected between the two variables (*P* > 0.05) (Table 3).

About 175 (36.2%) of the study participants were engaged in both low-intensity activity tasks and moderate-intensity activity, and the rest 134 (27.7%) were engaged in high-intensity activity. Larger proportions, 12 (6.9%), of the obese were engaged in moderate-intensity activity and one (0.6%) was on sedentary activity (Table 3).

### 3.3. Prevalence of Hypertension and Diabetes Mellitus with Obesity

With regard to the smoking status, there was no significant association between obesity and current or previous (who have stopped smoking) smoking habits. From 362 (74.8%) of the study participants who have had their blood pressure measured, 44 (12.2%) were hypertensive.

From the total obesity in the study population, 4 (9.1%) were hypertensive according to the report by the respondents. From 227 (46.9%) of the study participants who have had their blood sugar level measured, 21 (9.3%) were diabetic. From the total obese study participants, one (4.8%) was diabetic. Hypertension and obesity were associated in the study population (*P* < 0.05), but there was no association detected between obesity and diabetes mellitus (Table 4).

### 3.4. Factors Associated with Obesity

From the multivariable logistic regression analysis, history of hypertension, sex, and educational status were significantly associated with being overweight and obese. Nonhypertensive individuals were 86% less likely to be obese when compared to hypertensive (AOR = 0.14 (95% CI: 0.03–0.69)) (Table 4). Males were 90% less likely to be obese when compared to females (AOR = 0.10 (95% CI: 0.01–0.84)). Illiterate people were 94% less likely to be obese compared to those who were literate people (AOR = 0.06 (95% CI: 0.01–0.44)) (Table 5).

## 4. Discussion

A community-based cross-sectional study was conducted in Addis Ababa residents to determine the prevalence and associated factors of overweight/obesity. The prevalence of obesity is an emerging problem in Ethiopia. In the setting of the study population, female sex, educational status, and hypertension were found to be significantly associated with overweight and obesity. In this study, female sex, educational status, and hypertension were found to be significantly associated with overweight and obesity. In the current finding, women were more likely to be overweight/obese as compared to men. The current study is similar with the studies conducted in Ghana and Ethiopia [24, 25]. This might be due to the fact that women have more adipose tissues than men, and they are also exposed to more sedentary life style, and high-energy foods are usually consumed. The current study showed that literate people were more likely to be obese than uneducated people. According to the national DHS, overweight/obesity increases with education level. Similarly, those who have more than secondary education were four times as likely to be overweight or obese as compared with noneducated [9, 26]. This could be due to the fact that educated people are likely to consume better intake of high-fat and high-calorie diet and follow a sedentary life style, so they increase to be overweight and obese. In this study, the presence of hypertension was associated with obesity. Most studies have investigated the relationship between obesity and hypertension. People with obesity are more likely to have hypertension. Obesity is also associated with an increased cardiovascular risk and earlier onset of morbidity. The growing obesity emergence is one of the major sources of unsustainable health costs and morbidity and mortality due to hypertension [11, 27].

The study was community-based approach, and it may represent the prevalence of problem in the community. This study is based on the reported morbidities but not based on the results from laboratory or clinical diagnosis. There may be recall bias in reporting different food groups consumed over the previous day in the dietary diversity score. The study also does not assess the daily caloric intake of the study participants which could have its effect on obesity. The physical activity tool was not validated for the study population and may have under- or overestimated the main findings.

## 5. Conclusions

This study revealed an emerging problem of a high prevalence of overweight and obesity among adults of Addis Ababa residents at which the combined prevalence of overweight and obesity was found to be considerably high in Addis Ababa city residents compared to the national figure. Independent predictors of overweight/obesity in the population studied were female sex, educational status, and presence of hypertension. Various health sectors should give special emphasis to women, educated people, and prevention of hypertension as the problem of obesity prevails. They should have to enlighten the educated people on the importance of active lifestyle and regular physical exercise for the maintenance of good body size and health. In general, a thorough nutrition education or behavior change communication on obesity and its consequences has to be addressed to the study population. In addition, the concerned bodies should initiate efforts to tackle the newly emerging public health problem of the country and promote healthy lifestyle behaviors in the inhabitants of city settings.

## Figures and Tables

**Table 1:** The sociodemographic characteristics of the adult population in Addis Ababa, Ethiopia.

Variables (*n* = 484)	Frequency	Percent
*Sex*		
Male	253	52.3
Female	231	47.7

*Age groups*		
25–34	268	55.4
35–44	150	31
45–54	42	8.7
55–64	24	5

*Religion*		
Orthodox	361	74.6
Protestant	71	14.7
Muslim	47	9.7
Other	5	1

*Ethnicity*		
Amhara	259	53.5
Oromo	117	24.2
Tigre	45	9.3
Gurage	35	7.2
Wolaita	22	4.5
Others	6	1.2

*Marital status*		
Married	268	55.4
Single	184	38
Divorced	20	4.1
Widowed	12	2.5

*Educational status*		
No school	16	3.3
Elementary (1–6)	43	8.9
Junior (7-8)	29	6
High school (9–12)	59	12.2
College and above	337	69.6

*Occupation*		
Housewife	37	7.6
Merchant	146	30.2
Government employee	179	37
Non-government employee	80	16.5
Others	42	8.7

*Monthly income (birr)*		
<8000	277	57.2
≥8000	207	42.8

*Wealth index tercile (HH)*		
Low	170	35.1
Medium	167	34.5
High	147	30.4

**Table 2:** Prevalence of obesity by sociodemographic characteristics of study participants in Addis Ababa, Ethiopia.

Variables (*n* = 484)	Nutritional status (*N* = 14)		*P* value
	Obese	Nonobese	
	Number (%)	Number (%)	
*Age groups*			
25–34	3 (1.1)	265 (98.9)	0.007
35–44	5 (3.3)	145 (96.7)	
45–54	4 (9.5)	38 (90.5)	
55–64	2 (8.3)	22 (91.7)	

*Sex*			
Male	1 (0.4)	252 (99.6)	0.001
Female	13 (5.6)	218 (94.4)	

*Marital status*			
Married	10 (3.7)	258 (96.3)	<0.0001
Single	0 (0)	184 (100)	
Divorced	2 ()(10)	18 (90)	
Widowed	2 (16.7)	10 (83.3)	

*Family size*			
≤4	9 (2.6)	335 (97.4)	>0.05
>4	5 (3.6)	135 (96.4)	

*Occupation*			
Housewife	2 (5.4)	35 (94.6)	0.034
Merchant	9 (6.2)	137 (93.8)	
GE	2 (1.1)	177 (98.9)	
Non-GE	1 (1.3)	79 (98.8)	
Other	0 (0)	42 (100)	

*Monthly income*			
<8000 birr	5 (2.1)	230 (97.9)	>0.05
≥8000 birr	9 (4.3)	198 (95.7)	

*Have car*			
No	7 (2)	349 (98)	0.043
Yes	7 (5.5)	121 (94.5)	

*Wealth index (SES)*			
Low	1 (0.6)	169 (99.4)	0.003
Medium	3 (1.8)	164 (98.2)	
High	10 (6.2)	137 (93.2)	

GE = government employee. *SES* = socioeconomic status. *P* values refer to the chi-squared test for the difference observed between obese and nonobese participants.

**Table 3:** Prevalence of obesity by sex with age group, educational status, dietary diversity score, alcohol consumption, and total physical activity level among adults in Addis Ababa, Ethiopia.

Variables (*n* = 484)	Nutritional status (*N* = 14)		*P* value
	Obese	Nonobese	
	Number (%)	Number (%)	
*Male*			
25–34	0 (0)	125 (100)	>0.05
35–44	1 (1.1)	88 (98.9)	
45–54	0 (0)	19 (70.4)	
55–64	0 (0)	6 (50)	

*Female*			
25–34	3 (2.1)	140 (97.9)	>0.05
35–44	4 (6.6)	57 (93.4)	
45–54	4 (26.7)	11 (73.3)	
55–64	2 (16.7)	10 (83.3)	

*Educational status*			
No school	4 (25)	12 (75)	<0.0001
Elementary	0 (0)	43 (100)	
Junior	0 (0)	29 (100)	
High school	1 (1.7)	58 (98.3)	
College and above	9 (2.7)	328 (97.3)	

*Dietary diversity score*			
Low (0–3)	0 (0)	36 (100)	>0.05
Medium (4–5)	2 (2)	97 (98)	
High (≥6)	12 (3.4)	337 (96.6)	

*Total physical activity level (TPAL)*			
Highly active	1 (0.7)	133 (99.3)	<0.0001
Moderately active	12 (6.9)	163 (93.1)	
Sedentary	1 (0.6)	174 (99.4)	

*Average daily alcohol consumption*			
≤1 beer a day	9 (5.5)	154 (94.5)	>0.007
>1 beer a day	0 (0)	128 (100)	

*Currently consumed alcohol*			
Yes	9 (3)	290 (97)	>0.05
No	5 (2.7)	180 (97.3)	

*P* values refer to the chi-squared test for the difference observed between obese and nonobese participants.

**Table 4:** Prevalence of hypertension and diabetes mellitus in adults of Addis Ababa who have had their blood sugar level and blood pressure measured by obesity.

Variables	Nutritional status (*N* = 14)		COR (95% CI)	AOR (95% CI)
	Obese	Nonobese		
	Number (%)	Number (%)		
*Hypertension*				
No	8 (2.5)	310 (97.5)	0.26 (0.07–0.89)^*∗*^	0.14 (0.03–0.69)^*∗*^
Yes	4 (9.1)	40 (90.9)	1.00	1.00
Total	12 (3.3)	350 (96.7)	—	—

*Taking hypertensive drugs*				
No	10 (3)	322 (97)	—	>0.05
Yes	2 (6.7)	28 (93.3)	—	—

*Diabetes mellitus (DM)*				
No	9 (4.4)	197 (95.6)	0.91 (0.11–7.59)	—
Yes	1 (4.8)	20 (95.2)	1.00	—
Total	10 (4.4)	217 (95.6)	—	—

*Taking DM drugs*				
No	9 (4.2)	203 (95.8)	—	>0.05
Yes	1 (7.7)	12 (92.3)	—	—

^*∗*^ *P* < 0.05.

**Table 5:** Multivariable logistic regression model predicting obesity among adults in Addis Ababa, Ethiopia.

Variables (*n* = 484)	Status (*N* = 14)		Crude OR (95% CI)	Adjusted OR (95% CI)
	Obese	Nonobese		
	Number (%)	Number (%)		
*Age groups*				
25–34	3 (1.1)	265 (98.9)	8.03 (1.27 – 50)^*∗*^	3.99 (0.52–30)
35–44	5 (3.3)	145 (96.7)	2.64 (0.48 – 14)	1.60 (0.24 – 10)
45–54	4 (9.5)	38 (90.5)	0.86 (0.15–5)	0.92 (0.11–7)
55–64	2 (8.3)	22 (91.7)	1.00	1.00

*Sex*				
Male	1 (0.4)	252 (99.6)	0.07 (0.01 – 0.51)^*∗∗*^	0.10 (0.01–0.84)^*∗*^
Female	13 (5.6)	218 (94.4)	1.00	1.00

*Educational status*				
Illiterate	4 (25)	12 (75)	0.07 (0.02–0.24)^*∗∗*^	0.06 (0.01–0.44)^*∗∗*^
Literate	10 (2.1)	458 (97.9)	1.00	1.00

*Have car*				
No	7 (2)	349 (98)	0.35 (0.12–1.01)	—
Yes	7 (5.5)	121 (94.5)	1.00	—

*Total physical activity level*				
Highly active	1 (0.7)	133 (99.3)	0.08 (0.10–0.61)^*∗*^	0.17 (0.02–1.54)
Moderately active	12 (6.9)	163 (93.1)	0.76 (0.05–12)	1.11 (0.06–20)
Sedentary	1 (0.6)	174 (99.4)	1.00	1.00

*Marital status*				
Married	10 (3.7)	258 (96.3)	0.49 (0.15–1.57)	—
Unmarried	4 (1.9)	212 (98.1)	1.00	—

*Monthly income*				
<8000 birr	5 (2.1)	230 (97.9)	2.1 (0.69–6)	—
≥8000 birr	9 (4.3)	198 (95.7)	1.00	—

*Wealth index*				
Low	1 (0.6)	169 (99.4)	12.34 (1.56–97)^*∗*^	5.36 (0.47–61)
Medium	3 (1.8)	164 (98.2)	3.99 (1.08–14)	6.1 (0.98–37)
High	10 (6.2)	137 (93.2)	1.00	1.00

^*∗∗*^ *P* < 0.01; ^*∗*^*P* < 0.05.

## Data Availability

The data used to support the findings of this study are available from the corresponding author upon request.
